# Congenital dislocation of the knee complicated with bilateral hip dislocation: a case report and literature review

**DOI:** 10.1186/s12891-024-07316-1

**Published:** 2024-04-24

**Authors:** Bohai Qi, Qiang Jie, Xiaowei Wang, Qingda Lu, Fei Su, Yating Yang

**Affiliations:** 1 Pediatric Orthopaedic Hospital, Honghui Hospital of Xi’an Jiaotong University, Xi’an, China; 2 Xi’an Key Laboratory of Skeletal Deformity and Injury Repair, 555 Youyi East Road, Beilin District, Xi’an, Shaanxi 710021 China

**Keywords:** Congenital knee dislocation, Classification, Treatment

## Abstract

**Background:**

Congenital dislocation of the knee is characterised by excessive knee extension or dislocation and anterior subluxation of the proximal tibia, and this disease can occur independently or coexist with different systemic syndromes. Nevertheless, significant controversy surrounds treating this disease when combined with hip dislocation. This paper presents a case of a 4-month-old patient diagnosed with bilateral hip dislocation combined with this disease. The study discusses the pathophysiology, diagnosis, and treatment methods and reviews relevant literature.

**Case presentation:**

We reported a case of a 4-month-old female infant with congenital dislocation of the right knee joint, which presented as flexion deformity since birth. Due to limitations in local medical conditions, she did not receive proper and effective diagnosis and treatment. Although the flexion deformity of her right knee joint partially improved without treatment, it did not fully recover to normal. When she was 4 months old, she came to our hospital for consultation, and we found that she also had congenital dislocation of both hip joints and atrial septal defect. We performed staged treatment for her, with the first stage involving surgical intervention and plaster orthosis for her congenital dislocation of the right knee joint, and the second stage involving closed reduction and plaster fixation orthosis for her congenital hip joint dislocation. Currently, the overall treatment outcome is satisfactory, and she is still under follow-up observation.

**Conclusions:**

Early initiation of treatment is generally advised, as nonsurgical methods prove satisfactory for mild cases. However, surgical intervention should be considered in cases with severe stiffness, unresponsive outcomes to conservative treatment, persistent deformities, or diagnoses and treatments occurring beyond the first month after birth.

## Background

Congenital dislocation of the knee (CDK) is an uncommon congenital malformation. The clinical manifestations of this disease were initially documented by a 19th-century French surgeon, Guillaume Dupuytren, in 1834, identifying it as a condition characterized by congenital knee dislocation. The estimated incidence of this condition is approximately 1/100,000 [1, 2]; prenatal ultrasound can confirm the diagnosis at approximately 20 weeks of gestation [3].

## Case presentation

**Fig. 1:**
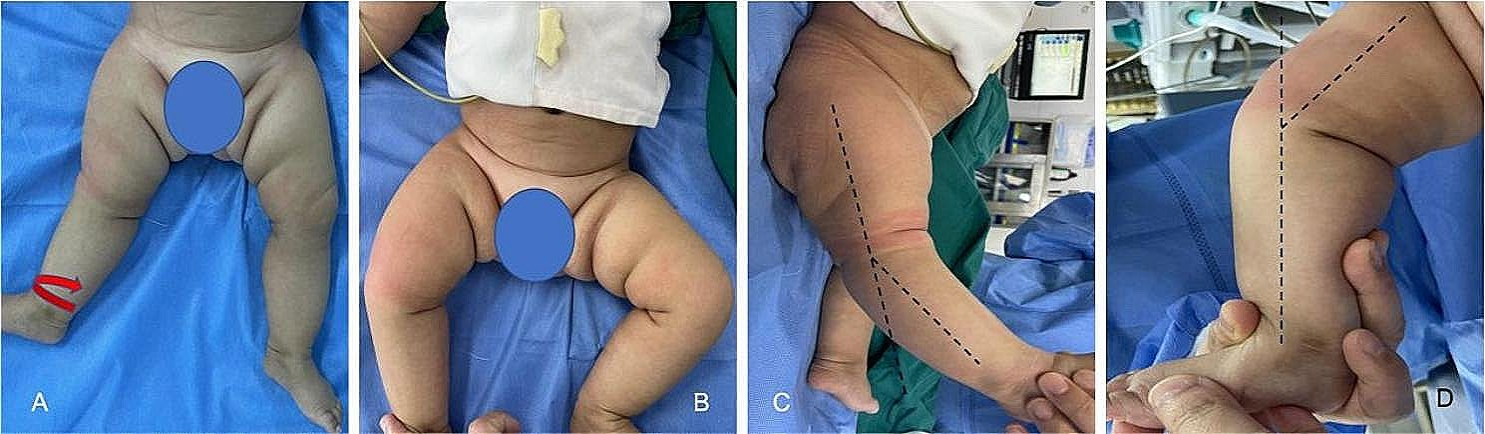
Photos documenting the appearance of the extremities. The right tibia exhibits external rotation of approximately 90° (**A** and **B**), while the right knee presents hyperextension of approximately 30° (**C**) and flexion of approximately 45° (**D**)

The patient is a female infant aged 4 months and 17 days, presenting limited movement and reverse flexion of the right knee joint since birth. On physical examination, bilateral asymmetry was observed in the thigh folds, approximately equal length of lower extremities, and bilateral prominent tuberosity of the femur moving outward and upward in the posterior iliac region. Both hips demonstrated an adduction and abduction range of approximately 30°-0°-45°, with regular flexion and extension activities. Ottolani’s and Barlow’s signs were negative in both hips. The right knee exhibits hyperextension, external rotation deformity, and notable soft tissue contracture. There was no local redness, swelling, or tenderness. A stress test on both knees yields negative results for inside and outside movements. There is an observable restriction in proper knee movement, with a range of motion showing flexion of 45°, dorsal extension of -30°, and external rotation of 90°. The left knee and ankle joint display normal movement, as does bilateral toe activity (Fig. 1). Laboratory tests showed normal results.

**Fig. 2:**
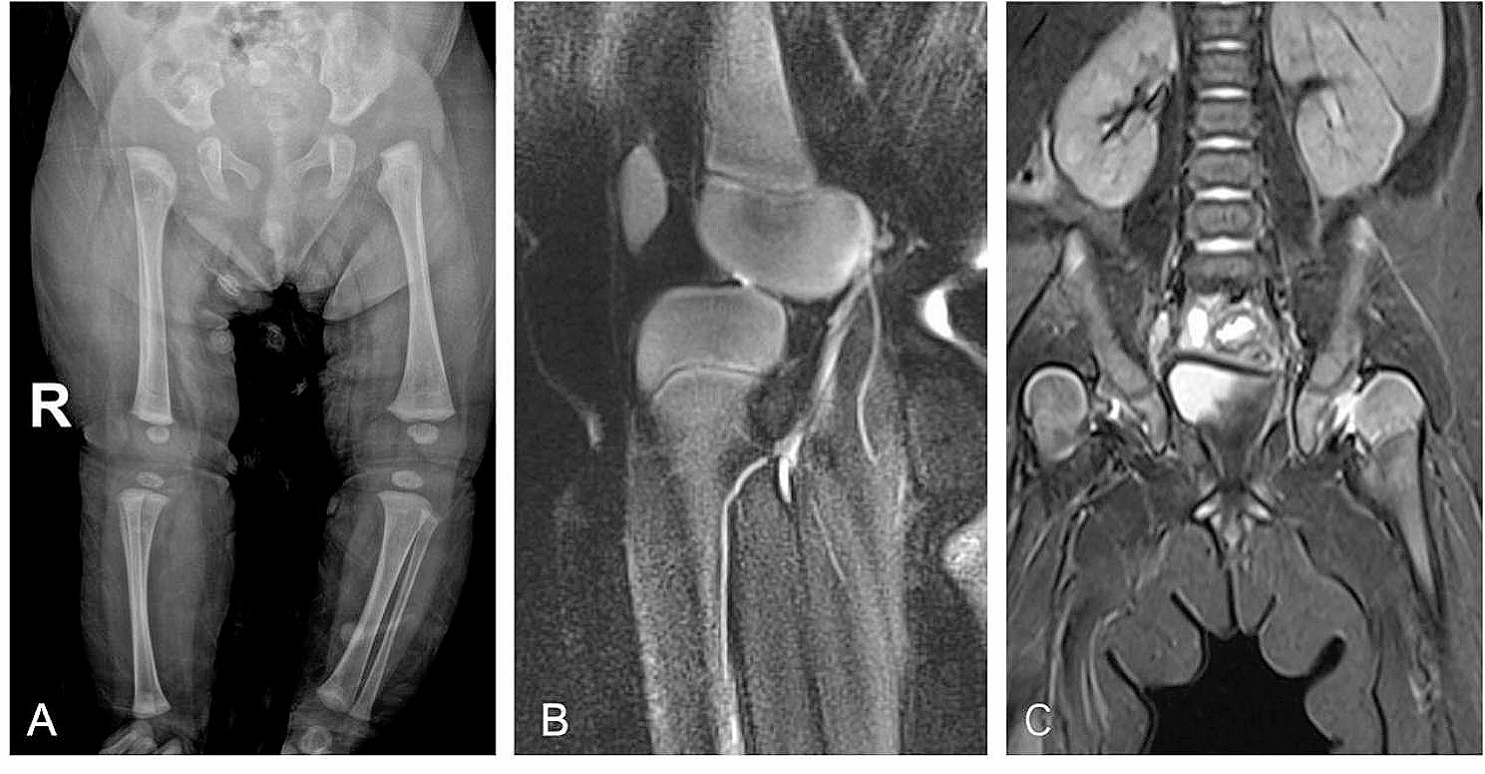
The full-length X-ray of the lower extremity revealed subluxation of the right knee joint, anterior shifting of the tibia, and external rotation (**A**). MRI of the right knee displayed significant anterior movement of the tibia in the right knee joint and a low and flat anterior cruciate ligament (**B**). MRI of the hip joint demonstrated superficial and flat bilateral acetabular development, with the bilateral femoral heads fully detached and displaced outward from the acetabular fossa (**C**)

**Fig. 3:**
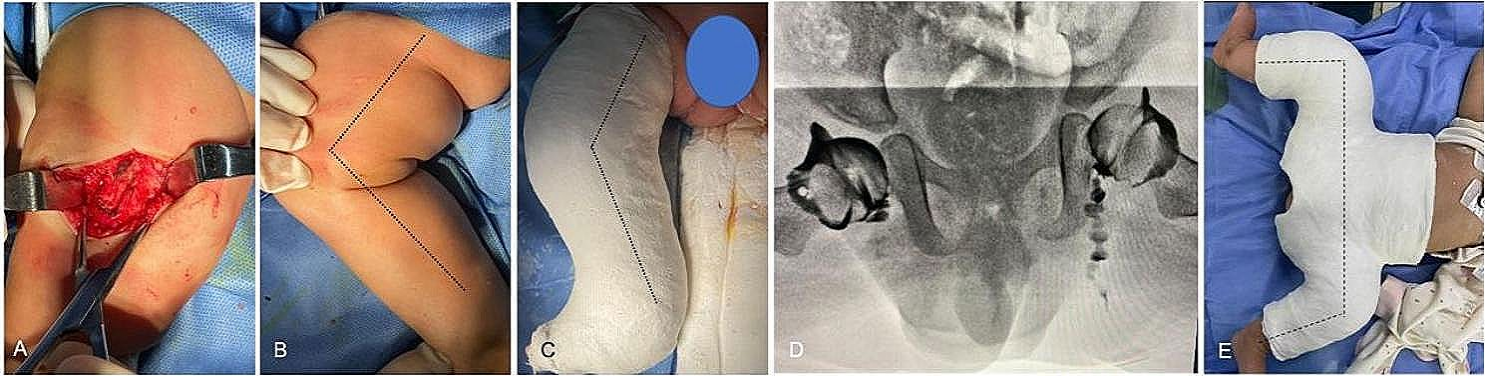
A longitudinal incision was made directly anterior to the distal right thigh, enabling the release and V-Y lengthening of the quadriceps muscle (**A**). Following quadriceps lengthening, the right knee achieved passive flexion of 90° (**B**). The right knee joint was repositioned with the proximal tibia in good alignment with the distal femur, and the right lower limb was immobilized in a tubular cast after flexion of the knee at 83°. (**C**); Removal of the cast at five weeks postoperatively, with the right knee flexed close to 90°, in closed resurfacing of bilateral developmental hip dislocation (**D**); Increase the angle of flexion of the right knee by approximately 20° and continue cast immobilization. (**E**)

Right knee joint ultrasound, lower extremity X-rays, and MRI of the right knee revealed right knee dislocation (Fig. 2A-B). MRI of the hip demonstrated bilateral hip dislocation (Fig. 2C). Cardiac ultrasound shows the presence of congenital heart disease, including an atrial septal defect. Diagnosis upon admission: (1) Type 2 congenital right knee dislocation, (2) Bilateralcongenital dislocation of the hip(CDH), (3) Congenital heart disease. Right quadriceps muscle lengthening (V-Y plasty), knee dislocation reduction, and postoperative right knee flexion 83° cast-type casting were performed under general anaesthesia (Fig. 3). The cast was removed five weeks after surgery, the flexion of both knees was close to 90°, and the flexion angle of the right knee was increased to continue fixation during bilateral hip dislocation closed reduction cast. The patient underwent two hip cast replacements over a period of four months in order to achieve hips stability and promote acetabular development. No specific complications were observed during the entire treatment course.Presently, the patient has been undergoing follow-up for 14 months and has begun learning to walk independently. It is worth noting that the right dislocation of knee has significantly cured. Despite the successful reduction of both hips, residual dysplasia of hip remains present. Overall, the treatment has produced satisfactory outcomes with no observed recurrence. Subsequently, further follow-up will be conducted.

## Discussion

**Fig. 4:**
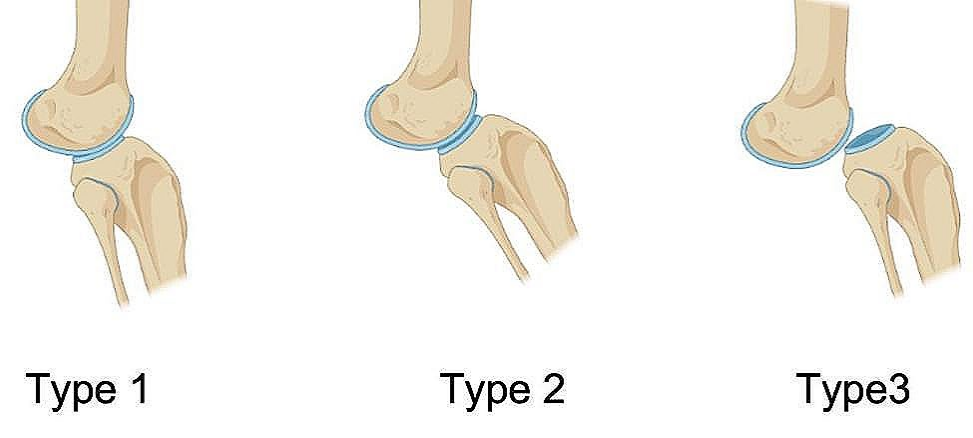
Note: Type 1: congenital knee hyperextension; Type 2: congenital knee subluxation; Type 3: congenital knee dislocation

There is currently some controversy regarding the classification of CDK; in earlier studies [4–6], CDK was classified into three types by scholars based on the anatomical relationship between the distal femur and proximal tibia: type 1 represents congenital knee hyperextension, type 2 involves congenital knee hyperextension combined with anterior subluxation of the tibia relative to the femur, and type 3 encompasses congenital knee hyperextension deformity accompanied by complete anterior dislocation of the tibia relative to the femur (Fig. 4). Following birth, the diagnosis can be confirmed by assessing the distinctive deformity appearance and conducting standard lateral knee radiographs. Furthermore, CDK frequently presents with additional skeletal abnormalities in the limbs, including hip dysplasia or dislocation [7–9]. There is another method of classification based on the degree of knee flexion. When the degree of knee flexion exceeds 90°, it is classified as Type I; when the degree of flexion is between 30° and 90°, it is classified as Type II; and when the degree of flexion is less than 30°, it is classified as Type III [10].

Moreover, Mehrafshan et al. [1] introduced a classification method that categorizes CDK into three types based on the criteria of reducibility and stability. Type I represents cases that are easily reducible and exhibit flexion stability, while Type II corresponds to stubborn dislocations that are reducible but unstable. Type III refers to irreducible dislocations.

Based on the anatomical relationship between the femur and tibia, our case is categorized as Type II; however, considering the challenges in reduction and stability, it may fall into Type I.

The precise cause of CDK is currently unknown. However, contributing factors can be categorized into extrinsic factors resulting from abnormal intrauterine pressure caused by fetal malposition and intrinsic factors arising from genetic variations and neuromuscular imbalances [11–13]. These viewpoints are supported by the consistent identification of quadriceps fibrosis in all cases. Bilateral CDK is predominantly linked to associated syndromes, including Larsen’s syndrome, Beals syndrome, Marfan’s syndrome, Ehlers‒Danlos syndrome, and myelomeningocele [1, 13, 14]. Several studies have reported that the likelihood of concurrent ipsilateral developmental hip dysplasia is 70%, while the probability of coexisting congenital clubfoot malformation is 50% [15–17].

Previous studies have indicated that isolated CDK is a rare anomaly, and most isolated cases exhibit a favorable prognosis, with excellent motor function of the lower limbs and ambulatory ability. Conversely, complex cases involving genetic syndromes or multiple anomalies generally have poor prognoses [18–20]. Moreover, there is a tendency for numerous malformations accompanied by developmental abnormalities affecting the upper extremities, face, digestive tract, and reproductive system. It is advisable to prioritize the treatment of CDK [19]. We report a case of unilateral CDK combined with bilateral CDH. In addition to the concurrent congenital atrial septal defect, no other abnormalities were identified. Hence, the potential accompanying syndrome has not been assessed, and no relevant genetic testing has been performed. In addition, we consider that hip dislocation may be congenital rather than developmental.

Research studies have indicated that the extent of ligament laxity in CDK cases is variable, particularly the cruciate ligament of the knee, which can be excessively elongated, shortened, or absent [15, 16, 21, 22]. Specifically, in cases associated with laxity-related syndromes, bilateral absence of the cruciate ligament in the knee is more prevalent. Conversely, in isolated cases of CDK, unilateral absence of the knee cruciate ligament is commonly observed, and the knee joint experiences gradual stabilization following joint dislocation reduction. CDK involves various pathoanatomical relationships, such as those related to the posterolateral and posteromedial knee structures, hamstring tendons, and anterior subluxation of the iliotibial band [10, 22]. In addition, there can be suprapatellar capsule atrophy, patellar dysplasia, and adhesions of the iliotibial band. Based on the combination of preoperative MRI and intraoperative observations, our case illustrates patellar infrapatellar contracture, adhesion of the iliotibial band, and shortening of the cruciate ligaments in the knee joint.

Before birth, the diagnosis can be confirmed through prenatal 3D or 4D ultrasound, typically performed around the 20th week of gestation [3]. By employing prenatal diagnosis, swiftly referring to adept pediatric orthopedic surgeons, and initiating early conservative treatment, the necessity of surgery can be significantly reduced and the efficacy of the treatment enhanced [18]. Following birth, the diagnosis is typically confirmed based on observing distinctive deformities, a physical examination, and conventional knee X-rays [23]. However, Type 1 (Type 2 in the reported case) may lack identifiable findings on X-rays, necessitating caution in the diagnosis [4].

Nonsurgical treatment is recommended for newborn cases whenever possible. Nonsurgical treatment involves gentle manual reduction and the application of continuous casting, starting with knee extension and gradually transitioning to knee flexion. The cast was changed every two weeks for 2–3 months. In cases of combined CDH, the Pavlik harness can be utilized concurrently [24]. The treatment should be prioritized in cases involving concurrent hip and foot deformities. This prioritization can lead to improved outcomes for hip and foot deformities [19].

Surgical intervention is typically deemed necessary when nonsurgical treatment proves ineffective, and it is crucial to complete the surgical procedure between 1.5 and 2 years of age [21]. However, there is a belief among some doctors that surgical intervention should occur before the baby initiates standing, ideally at approximately six months of age [4]. The classic procedure involves V-Y lengthening of the quadriceps muscle and release of the anterior joint capsule and surrounding adhesion tissue, enabling the affected knee joint to achieve a partial range of motion that may not be entirely normal [6]. Osteotomy of the femur or tibia may be necessary in confident older children. Furthermore, an alternative treatment approach has been proposed by another physician, involving the minimally invasive release of the quadriceps muscle through a small incision and subsequent casting. While short-term follow-up has shown promising corrective potential, the lack of long-term follow-up limits the assessment of its efficacy [25]. After a comprehensive evaluation, we used a series of soft tissue releases, such as quadriceps V-Y lengthening and plaster fixation, to achieve the orthopedic goal. To date, there is an effective correction of knee dislocation, a flexion angle of more than 90°, and no signs of redislocation.

CDK is a rare condition that typically exhibits a favorable prognosis in isolated cases but presents a generally poor prognosis when accompanied by multiple deformities [18]. However, a retrospective study involving 9 patients with at least 9 years of follow-up ,it was demonstrated that timely diagnosis and prompt initiation of suitable conservative treatment resulted in favorable long-term functional outcomes in the affected limbs, with few occurrences of complications and recurrence [26]. A retrospective study was conducted on 24 patients who received surgical treatment more than six months after birth. The knee was dislocated in 45 limbs; the hip was dislocated in 40 instances. It revealed that most cases with concurrent dislocation of the knee and hip joints achieved positive treatment outcomes through surgical intervention. Notably, patients presenting with ligament laxity exhibited even better efficacy. Furthermore, in a small number of cases, spontaneous reduction of hip dislocation was observed following corrective surgery for knee dislocation and subsequent recovery of knee flexion function [27].

## Conclusion

We present a case report of delayed diagnosis with unilateral CDK (right) combined with bilateral CDH. The patient achieved favorable outcomes following surgical intervention for right knee.She is undergoing closed reduction and immobilization using a hip spica orthosis to address the bilateral hip dislocation. CDK can be readily detected in affected children at birth, as consistently reported by most researchers. Timely intervention proves effective in managing this condition. A comprehensive assessment of the newborn and any accompanying deformities upon delivery holds utmost importance, particularly in evaluating isolated or syndromic needs, the severity of knee dislocation, and the level of stiffness—such evaluations aid in determining the appropriate treatment approach, encompassing conservative and surgical interventions. Early initiation of treatment is generally advised, as nonsurgical methods prove satisfactory for mild cases. However, surgical intervention should be considered in cases with severe stiffness, unresponsive outcomes to conservative treatment, persistent deformities, or diagnoses and treatments occurring beyond the first month after birth.

## Data Availability

Data are available for review.

## Abbreviations

CDK: Congenital dislocation of the knee
CDH: Congenital dislocation of the hip
MRI: Magnetic Resonance Imaging
